# Impact of lower-respiratory tract infections on healthcare utilization and mortality in older adults: a Swedish population-based cohort study

**DOI:** 10.1007/s40520-024-02808-5

**Published:** 2024-07-17

**Authors:** Ahmad Abbadi, Susanna Gentili, Eleana Tsoumani, Agnes Brandtmüller, Merle K. Hendel, Stina Salomonsson, Amaia Calderón-Larrañaga, Davide L. Vetrano

**Affiliations:** 1 Aging Research Center, Department of Neurobiology, Care Sciences and Society, Karolinska Institutet and Stockholm University, Stockholm, Sweden; 2 Department of Medical Epidemiology and Biostatistics, Karolinska Institutet, Nobels väg 12A, Solna, 171 65 Stockholm, Sweden; 3 Center for Observational and Real-World Evidence, MSD, Athens, Greece; 4 Center for Observational and Real-World Evidence, MSD, Budapest, Hungary; 5 Center for Observational and Real-World Evidence, MSD, Stockholm, Sweden; 6 Stockholm Gerontology Research Center, Stockholm, Sweden

**Keywords:** Lower respiratory tract infections, Older adults, Mortality, Healthcare use, Sex stratification

## Abstract

**Background:**

Lower respiratory tract infections (LRTIs) have an immediate significant impact on morbidity and mortality among older adults. However, the impact following the infectious period of LRTI remains understudied. We aimed to assess the short- to long-term impact of LRTIs on hospitalization, mortality, and healthcare utilization in older adults.

**Methods:**

Data from the Swedish National Study of Aging and Care in Kungsholmen (SNAC-K) was analyzed, with data from 2001 to 2019 for mortality and 2001–2016 for healthcare utilization. LRTI-exposed participants were identified and matched with LRTI-nonexposed based on sociodemographics, lifestyle factors, and functional and clinical characteristics. Statistical models evaluated post-LRTI hospitalization risk, days of inpatient hospital admissions, healthcare visits, and mortality.

**Results:**

567 LRTIs-exposed participants during the study period and were matched with 1.701 unexposed individuals. LRTI-exposed individuals exhibited increased risk of hospitalization at 1-year (HR 2.14, CI 1.74, 2.63), 3-years (HR 1.74, CI 1.46, 2.07), and 5-years (HR 1.59, CI 1.33, 1.89). They also experienced longer post-LRTI hospital stays (IRR 1.40, CI 1.18, 1.66), more healthcare visits (IRR 1.47, CI 1.26, 1.71), specialist-care visits (IRR 1.46, CI 1.24, 1.73), and hospital admissions (IRR 1.57, CI 1.34, 1.83) compared to nonexposed participants over 16-years of potential follow-up. Additionally, the 19-year risk of mortality was higher among LRTI-exposed participants (HR 1.45, CI 1.24, 1.70). Men exhibited stronger associations with these risks compared to women.

**Conclusions:**

LRTIs pose both short- and long-term risks for older adults, including increased risks of mortality, hospitalization, and healthcare visits that transpire beyond the acute infection period, although these effects diminish over time. Men exhibit higher risks across these outcomes compared to women. Given the potential preventability of LRTIs, further public health measures to mitigate infection risk are warranted.

**Supplementary Information:**

The online version contains supplementary material available at 10.1007/s40520-024-02808-5.

## Introduction

Lower respiratory tract infections (LRTIs) –among them pneumonia– are major causes of mortality and morbidity globally [1–3]. LRTIs occur primarily within children under 5 and older adults above 70 [1–4]. Mortality immediately following LRTIs is highest among older adults and males [4]. Understanding the complexity of respiratory infections and their short- to long-term consequences beyond the acute infectious period on healthcare utilization is of utmost importance to properly plan and implement effective preventive strategies [4–6].

The burden of LRTIs is not limited to immediate mortality, as hospital care, treatments, and length of stay are clinical and public health concerns [4]. Although mild infections can resolve within a few days, more severe infections can take longer to achieve full functional recovery, which might reverberate in an increased healthcare utilization in the years following the infectious episode [7–9]. The impact of a single episode of LRTI in the geriatric population on long-term outcomes following the infection recovery remains under-researched [10].

Existing evidence suggests that LRTIs are associated with health consequences that persist beyond the duration of the infection itself. For example, reversible cognitive decline can last up to 2.5 years following the initial episode [10]. It is also reported that hospital length of stay is longer among males during the infection episode [11–14], and that obesity is associated with higher infection risk [15], but the roles of sex and obesity on the risk of mortality following the acute phase of pneumonia and LRTIs remain under debate [16]. Furthermore, the impact of LRTIs on future hospitalizations and their duration, as well as utilization of outpatient specialized healthcare remains largely understudied.

Therefore, in this study we aimed: (1) to estimate the impact of LRTIs on the risk of mortality and healthcare utilization post active infection period of LRTIs among older adults; (2) to examine the moderating effects of sex, age, and obesity on the aforementioned associations.

## Methods

### Study design

A population-based matched cohort study was performed using the Swedish National Study of Aging and Care in Kungsholmen (SNAC-K), capturing episodes of LRTIs between 2001 and 2016. SNAC-K is a prospective cohort study that includes adults aged 60 and above living in Kungsholmen, Stockholm, Sweden [17]. The study selected the participants using stratified sampling based on their age. Adults aged less than 78 were grouped in 6-year age intervals, while those older were grouped in 3-year age intervals. Follow-up is done every 6 years among the younger age groups, and every 3 years for the older age groups. The baseline cohort (2001–2004) consisted of 3.363 participants. Data in SNAC-K is collected using interviews, physical examinations, and laboratory tests by doctors, nurses, psychologists. Collected data include, among others, on demographics, living conditions, health risk behaviors, type and number of diseases and medications, functional and cognitive measures, and disability. Additional information on SNAC-K can be found elsewhere [17].

The cohort was integrated with data from the Swedish National Patient Register, providing information on both inpatient care and specialized outpatient care in Sweden, capturing both secondary and tertiary care activity [18]. Additionally, data from the Swedish Cause of Death Register provided information on all deaths occurring in Sweden since 1952 [19]. End of follow-up was until December 2016 for healthcare utilization outcomes, and until December 2019 for mortality. The Swedish registers used in this study has been appraised for their high completion and validation [19, 20].

### Exposure

LRTIs were identified using the National Patient Register based on International Classification of Diseases, 10th revision (ICD-10) codes J09-J18 and J20-22 (Table S1). Any primary or secondary diagnosis in the register was considered as a LRTI episode. The exposure time was operationalized as the time of the first LRTI episode for each participant occurring after the SNAC-K baseline interview date, and no additional episodes were considered thereafter. The participants who did not experience LRTIs during the follow-up were considered unexposed. The registers only captured LRTI cases that required hospital admission or treatment by specialized outpatient care in secondary or tertiary setting. Henceforth, the LRTI cases captured represent arguably moderate and severe cases of LRTIs. No LRTI cases from primary care were collected.

### Outcomes

Mortality was captured from the Cause of Death Register. Only all-cause mortality was considered in our study. Post-LRTI healthcare utilization outcomes were identified from the National Patient Register. Post-LRTI healthcare utilization was considered for all-cause inpatient hospitalization (yes/no), hospital length of stay (days spent in the hospital), number of inpatient admissions to hospitals, and number of specialized outpatient care visits. Post-LRTI total healthcare visits included both numbers of inpatient hospital admissions and numbers of specialized outpatient care visits. The first episode of LRTI that could have resulted in an inpatient hospitalization or healthcare visit was not counted (diagnosis and/or treatment visit), and only future hospitalizations and specialized outpatient care visits were counted regardless of the cause of post-LRTI healthcare visit. The number of days spent in the hospital were captured by summing up all post-LRTI inpatient hospital days after 1-, 3- and 5-years, and the total post-LRTI days spent in the hospitals by summing up the days throughout the 16-years of follow-up (excluding first inpatient admission for diagnosis/treatment). Details about the operationalization of all outcomes are provided in Table S1.

### Covariates

Sociodemographic information comprised age (continuous), sex (male/female), level of education categorized by years of formal education (elementary: up to 9th grade, high school: from 10 to 12th grade, university or equivalent: more than 12 years of formal education), living arrangement (independent living or group living), and civil status (married, including cohabitation partnership; divorced, including separated partnership; unmarried; widowed). Lifestyle factors included alcohol intake categorized by levels of consumption (never/occasional, light/moderate, heavy), smoking status (never smoker, former smoker, current smoker), and body mass index (BMI, continuous). Clinical characteristics included the presence or absence of specific chronic conditions (asthma, atrial fibrillation, cerebrovascular disease, chronic kidney disease, chronic liver disease, chronic obstructive pulmonary disease (COPD), cancer, diabetes, heart failure, hypertension, ischemic heart disease), the count of medications taken (continuous), and the count of chronic diseases (continuous). Chronic diseases were defined based on the operationalization by Calderón-Larrañaga et al. [21], and identified from the Swedish National Patient Register and the SNAC-K study visits (i.e., based on a specific list of ICD-10 codes). Functional characteristics included physical function, cognitive function, mild disability, and severe disability. Physical function was assessed through the walking speed (gait speed) test, using the 6-m test or the 2.44-m test for those unable to complete the former. Values were standardized to meters/second. For participants unable to perform the test (e.g., wheelchair users), a score of zero was assigned. Cognitive function was evaluated using the mini-mental state examination (MMSE), with scores ranging from 0 (worst result) to 30 (best result). Mild disability was measured by counting the number of impaired instrumental activities of daily living (I-ADL), encompassing grocery shopping, meal preparation, housekeeping, laundry, managing money, using the telephone, taking medications, and using public transportation, with scores ranging from 0 to 8. Severe disability was assessed by counting the number of impaired personal activities of daily living (P-ADL), covering bathing, dressing, toileting, continence, transferring, and eating, with scores ranging from 0 to 6.

### Statistical analysis

Summary information on baseline characteristics was reported in terms of means and standard deviations and counts with percentages for categorical variables. Chi-square, t-test, ANOVA, and Kruskal–Wallis tests were used for testing differences in demographic and other characteristic variables between the exposed and unexposed.

The exposed and unexposed participants would have been followed up from different starting points due to the exposure to LRTIs. This would have resulted in a later starting time among those who were LRTI-exposed compared to those unexposed, leading to potential biases in the study. To overcome this obstacle, the beginning of follow-up time among the non-exposed was randomly generated using a dummy date (index date) variable on a date between the start and end of follow-up, simulating the same scenario of LRTI exposed participants [10]. Matching was performed using a propensity score based on the study’s covariates of interest (sociodemographic, lifestyle factors, chronic diseases, and functional characteristics). The value of the covariates was assigned based on the immediate follow-up measures right before the index dates. The matching utilized an optimal matching procedure using a 1:3 ratio (exposed: unexposed), and participants could not be matched again once chosen. This method was used previously and described in detail elsewhere [10]. This step was conducted to reduce the extreme measures among the unexposed by matching them to the closest characteristics to those exposed to LRTIs.

Due to potential statistical differences in covariates between the exposed and unexposed, inverse probability weighting (IPW) was used to reduce the post matching remaining confounding and emulate randomization between exposed and non-exposed participants. This was done by giving weights to each participant based on the likelihood to be exposed or not exposed to LRTIs [22, 23]. To generate the weights, the user-written STATA command stipw was used [24, 25].

Poisson regression models were used to compute 1-, 3-, 5-years rate of days spent in the hospital, and 16-years of potential follow-up for days spent in the hospital, total healthcare visits, total specialized outpatient visits, and total hospital admission risk ratios. For 19-years all-cause mortality and 1-, 3-, and 5-years time to first hospitalization, survival models were computed using Royston-Parmer flexible parametric models [26]. These models allow for variation in the degrees of freedom for the baseline hazard and construction of smoothed cumulative incidence functions (CIF). The choice of the degrees of freedom was done using the lowest Akaike’s Information Criteria (AIC) and Bayesian Information Criteria (BIC) [27]. The user-written STATA command stpm2 was used to perform the analysis [26]. Degrees of freedom were capped at 5-degrees for simplicity reasons. To mitigate the strong confounding effect of age on all-cause mortality and increase the comparability between the exposed and unexposed groups, age was used as the time scale in the analyses concerning risk of death. For hospital admissions, time of follow-up was used as the time scale. The analyses were stratified by sex (male/female), age (< 75, ≥ 75 years), and obesity (based on BMI into no obesity: BMI < 30, and obesity: BMI ≥ 30). STATA 16.1 was used to perform the analysis.

### Ethical considerations

Ethics Committee at Karolinska Institutet (KI) and the Regional Ethical Review Board in Stockholm have approved the SNAC-K study and its follow-ups including usage and linkage to national Swedish registers (Dnrs: 01–114, 04–929/3, 2007/279–31, 2009/595–32, 2010/447–31/2, 2013/828–31/3 and 2016/730–31/1). All participants gave their consent before participating in the study, and their participation was voluntary. A next of kin was interviewed in those cases where participants were cognitively impaired. Strengthening the reporting of observational studies in epidemiology (STROBE) checklist is available in Supplementary file 2.

## Results

Among the 3,363 individuals participating in the SNAC-K study, a total of 567 participants were LRTI-exposed over the 16-year follow-up period. These individuals were matched to 1,701 LRTI-unexposed participants. Table 1 provides a comprehensive overview of the participants' sociodemographic, lifestyle factors, and functional and clinical characteristics at baseline (based on index date), following the matching process. While 41.6% of the LRTI-exposed participants were male, 38.4% were so among the unexposed group. The average age of the unexposed group was 78.1 years (± 9.3), whereas the average age of those exposed to LRTIs was 82.6 years (± 9.3).

**Table 1:** Sociodemographic, functional, and clinical characteristics of the study sample stratified by LRTI status

	No LRTI	LRTI	Total
	N = 1.701	N = 567	N = 2.268
Age*	78.1 (9.3)	82.6 (9.3)	79.2 (9.5)
Sex (female)	1.048 (61.6%)	331 (58.4%)	1.379 (60.8%)
Education*			
Elementary	272 (16.0%)	112 (19.8%)	384 (16.9%)
High school	852 (50.1%)	295 (52.0%)	1.147 (50.6%)
University	577 (33.9%)	160 (28.2%)	737 (32.5%)
Living arrangement			
Independent	1.624 (95.5%)	538 (94.9%)	2.162 (95.3%)
Group living	77 (4.5%)	29 (5.1%)	106 (4.7%)
Civil status			
Married	701 (41.2%)	210 (37.0%)	911 (40.2%)
Widow/er	485 (28.5%)	191 (33.7%)	676 (29.8%)
Unmarried	278 (16.3%)	89 (15.7%)	367 (16.2%)
Divorced	237 (13.9%)	77 (13.6%)	314 (13.8%)
Alcohol intake*			
Never/occasional	621 (36.5%)	246 (43.4%)	867 (38.2%)
Light/moderate	722 (42.4%)	210 (37.0%)	932 (41.1%)
Heavy	358 (21.0%)	111 (19.6%)	469 (20.7%)
Smoking status			
Never	1.001 (58.8%)	351 (61.9%)	1.352 (59.6%)
Former	441 (25.9%)	131 (23.1%)	572 (25.2%)
Current	259 (15.2%)	85 (15.0%)	344 (15.2%)
BMI (kg/m^2^)	25.4 (4.3)	25.1 (4.2)	25.4 (4.3)
Number of drugs*	4.5 (3.4)	6.0 (4.1)	4.9 (3.7)
Number of chronic diseases*	5.1 (2.9)	7.0 (4.0)	5.6 (3.3)
Asthma*	123 (7.2%)	59 (10.4%)	182 (8.0%)
Atrial fibrillation*	188 (11.1%)	129 (22.8%)	317 (14.0%)
Cerebrovascular disease*	147 (8.6%)	94 (16.6%)	241 (10.6%)
Chronic kidney disease*	617 (36.3%)	279 (49.2%)	896 (39.5%)
Chronic liver disease	5 (0.3%)	1 (0.2%)	6 (0.3%)
COPD*	96 (5.6%)	74 (13.1%)	170 (7.5%)
Cancer*	187 (11.0%)	114 (20.1%)	301 (13.3%)
Diabetes	199 (11.7%)	106 (18.7%)	305 (13.4%)
Heart failure*	183 (10.8%)	137 (24.2%)	320 (14.1%)
Hypertension	1.293 (76.0%)	439 (77.4%)	1.732 (76.4%)
Ischemic heart disease*	270 (15.9%)	162 (28.6%)	432 (19.0%)
Walking speed (m/s)*	0.9 (0.4)	0.8 (0.4)	0.9 (0.4)
MMSE*	28.0 (2.8)	27.2 (3.5)	27.8 (3.0)
ADL*	0.7 (1.6)	1.2 (2.0)	0.8 (1.7)
IADL*	0.2 (0.6)	0.3 (0.8)	0.2 (0.7)

Data are presented as mean (SD) for continuous measures, and n (%) for categorical measures

*BMI* body mass index, *COPD* chronic obstructive pulmonary disease, *LRTI* lower respiratory tract infection, *MMSE* mini-mental state examination, *ADL* personal activities of daily living, *IADL* instrumental activities of daily living

*p-value < 0.05

Since the matching process failed to completely reduce the differences between the two groups (Table 1), IPWs were additionally used in the analysis to further account for confounding.

### All-cause mortality

Table 2 shows the hazard ratio (HR) of mortality following a LRTI. Maximum follow-up was up to 19 years, and Table S2 shows the HRs of baseline hazard and restricted cubic splines. The youngest participant was 60 years old at the start of follow-up, while the oldest by the end of follow-up was 108. Those exposed to LRTIs had 1.45 (95% CI 1.24, 1.70) higher hazards of mortality compared to unexposed participants following the initial infectious episode. In the stratified analyses, the higher hazard of all-cause mortality following LRTIs among participants aged ≥ 75 vs. those aged < 75 (HR 1.60, 95% CI 1.34, 1.89 vs. HR 1.06, 95% CI 0.75, 1.51) was higher. Regarding sex stratification, males had higher point estimate compared to females (HR 1.65, 95% CI 1.26, 2.15 vs. HR 1.37, 95% CI 1.13, 1.66) but overlapping confidence intervals.

**Table 2:** All-cause mortality^a^ among those exposed and unexposed to LRTIs

	Proportion (Events/Total)	IPW-weighted HR	95% CI
Overall			
No LRTI	957/1701	Ref	Ref
LRTI	419/567	1.45**	(1.24, 1.70)
Males			
No LRTI	350/653	Ref	Ref
LRTI	180/236	1.65**	(1.26, 2.15)
Females			
No LRTI	607/1048	Ref	Ref
LRTI	239/331	1.37**	(1.13, 1.66)
Age < 75			
No LRTI	216/678	Ref	Ref
LRTI	56/137	1.06	(0.75, 1.51)
Age ≥ 75			
No LRTI	741/1023	Ref	Ref
LRTI	363/430	1.60**	(1.34, 1.89)
No Obesity			
No LRTI	851/1475	Ref	Ref
LRTI	377/501	1.45**	(1.22, 1.71)
Obesity			
No LRTI	106/226	Ref	Ref
LRTI	42/66	1.44	(0.94, 2.22)

Bold values are statistically significant

*HR* hazard ratio, *IPW* inverse probability weight, *LRTI* lower respiratory tract infection, *ref* reference

^a^Up to 19-years of follow-up

^*^p-value < 0.05, ** p-value < 0.01

### Risk of hospitalization

Table 3 displays the overall and stratified association between LRTIs and hospitalization risk at 1-, 3-, and 5-years, and Table S3 shows the HRs of baseline hazard and restricted cubic splines. LRTI-exposed participants had a significantly higher hazard of hospitalization at 1-year (HR 2.14, 95% CI 1.74, 2.63), 3-years (HR 1.74, 95% CI 1.46, 2.07), and 5-years (HR 1.59, 95% CI 1.33, 1.89) compared to those who were not exposed to LRTIs. In stratified analyses, the association between LRTIs and hospitalization was stronger among males and participants with obesity at 1-, 3-, and 5-years, but the risk was decreasing with increased follow-up time. At 5-years, the 95% CI was completely non-overlapping between males (HR 2.17, 95% CI 1.70, 2.79) and females (HR 1.33, 95% CI 1.06, 1.68).

**Table 3:** Risk of hospitalization among those exposed and unexposed to LRTIs

	Proportion (Hospitalized/Total)	IPW-weighted HR	95% CI
1-year hospitalization			
Overall			
No LRTI	385/1701	Ref	Ref
LRTI	258/567	2.14**	(1.74; 2.63)
Males			
No LRTI	157/653	Ref	Ref
LRTI	118/236	2.84**	(2.08; 3.89)
Females			
No LRTI	228/1048	Ref	Ref
LRTI	140/331	1.77**	(1.35; 2.33)
Age < 75			
No LRTI	108/678	Ref	Ref
LRTI	46/137	2.26**	(1.49; 3.42)
Age ≥ 75			
No LRTI	277/1023	Ref	Ref
LRTI	212/430	2.45**	(1.97; 3.04)
No Obesity			
No LRTI	342/1475	Ref	Ref
LRTI	226/501	1.96**	(1.57; 2.45)
Obesity			
No LRTI	43/226	Ref	Ref
LRTI	32/66	3.95**	(2.22; 7.04)
3-year hospitalization			
Overall			
No LRTI	822/1701	Ref	Ref
LRTI	359/567	1.74**	(1.46; 2.07)
Males			
No LRTI	314/653	Ref	Ref
LRTI	162/236	2.35**	(1.80; 3.06)
Females			
No LRTI	508/1048	Ref	Ref
LRTI	197/331	1.45**	(1.15; 1.83)
Age < 75			
No LRTI	235/678	Ref	Ref
LRTI	84/137	2.06**	(1.50; 2.82)
Age ≥ 75			
No LRTI	587/1023	Ref	Ref
LRTI	275/430	2.03**	(1.69; 2.43)
No Obesity			
No LRTI	731/1475	Ref	Ref
LRTI	317/501	1.66**	(1.37; 2.00)
Obesity			
No LRTI	91/226	Ref	Ref
LRTI	42/66	2.43**	(1.48; 3.99)
5-year hospitalization			
Overall			
No LRTI	1051/1701	Ref	Ref
LRTI	388/567	1.59**	(1.33; 1.89)
Males			
No LRTI	402/653	Ref	Ref
LRTI	174/236	2.17**	(1.70; 2.79)
Females			
No LRTI	649/1048	Ref	Ref
LRTI	214/331	1.33*	(1.06; 1.68)
Age < 75			
No LRTI	333/678	Ref	Ref
LRTI	96/137	1.80**	(1.33; 2.42)
Age ≥ 75			
No LRTI	718/1023	Ref	Ref
LRTI	292/430	1.93**	(1.62; 2.31)
No Obesity			
No LRTI	927/1475	Ref	Ref
LRTI	343/501	1.52**	(1.26; 1.83)
Obesity			
No LRTI	124/226	Ref	Ref
LRTI	45/66	2.13**	(1.31; 3.47)

Bold values are statistically significant

*IPW* inverse probability weight, *LRTI* lower respiratory tract infection, *HR* hazard ratio, *ref* reference

^*^p-value < 0.05, ** p-value < 0.01

### Length of stay and total healthcare visits

Table 4 presents the findings of the Poisson regression models examining 1-, 3-, and 5-years, and total (throughout a maximum of 16-years follow-up) of days spent in hospital, total healthcare visits, total specialized outpatient visits, and total hospital admissions. The results indicate that LRTI-exposed participants had a higher rate of days spent in the hospital compared to non-LRTI exposed participants, a difference that decreased with time, with incidence rate ratios (IRR) of 2.12 at 1-year, 1.63 at 3-years, 1.47 at 5-years, and 1.40 at up to 16-years of follow-up. Across all models, LRTI-exposed participants had a higher incidence of healthcare utilization compared to LRTIs-unexposed participants. Supplementary tables (Tables S4-S10) provide further insights by stratifying the analyses by sex, age, and obesity. Males and adults aged < 75 had higher IRR of days spent in the hospital at all measured times. Meanwhile, no significant findings were obtained for females at 5-years or longer follow-up regarding number of days of inpatient hospital stay, nor for older adults (aged ≥ 75) regarding total specialized outpatient visits. Participants with vs without obesity had higher IRR point estimates in all healthcare utilization outcomes, but confidence intervals were overlapping.

**Table 4:** Rate of days stayed in inpatient hospital admissions and healthcare visits among those exposed and unexposed to LRTIs

	Mean ± SD	IPW-weighted IRR	95% CI
Overall length of stay (in days)^a^			
No LRTI	24.7 ± 34.0	Ref	Ref
LRTI	30.9 ± 45.3	1.40**	(1.18; 1.66)
1-year length of stay (in days)			
No LRTI	3.2 ± 10.7	Ref	Ref
LRTI	8.6 ± 17.0	2.12**	(1.62; 2.77)
3-year length of stay (in days)			
No LRTI	9.6 ± 19.4	Ref	Ref
LRTI	14.2 ± 26.1	1.63**	(1.33; 1.99)
5-year length of stay (in days)			
No LRTI	15.3 ± 26.0	Ref	Ref
LRTI	24.1 ± 35.9	1.47**	(1.23; 1.77)
Total healthcare visits^a^			
No LRTI	21.0 ± 23.3	Ref	Ref
LRTI	20.6 ± 40.5	1.47**	(1.26; 1.71)
Total specialized outpatient visits^a^			
No LRTI	18.2 ± 21.7	Ref	Ref
LRTI	16.9 ± 38.5	1.46**	(1.24; 1.73)
Total hospital admissions^a^			
No LRTI	2.2 ± 2.8	Ref	Ref
LRTI	3.2 ± 4.6	1.57**	(1.34; 1.83)

Bold values are statistically significant

*IPW* inverse probability weight, *IRR* incidence rate ratio, *LRTI* lower respiratory tract infection, *ref* reference

^a^Up to 16-years potential follow-up

^*^p-value < 0.05, ** p-value < 0.01

## Discussion

The aim of this study was to evaluate the risk of mortality and healthcare utilization in terms of time to admission to the hospital, days spent in the hospital, and number of hospital admissions and specialized outpatient care visits following the acute phase of a single episode of LRTI among older adults aged ≥ 60 years, excluding the initial hospital admission or specialized outpatient visit during the diagnosis and/or treatment of LRTI. The findings revealed important insights into the consequences of LRTIs among older adults. Firstly, LRTIs were associated with higher risk of all-cause mortality within a 19-years follow-up, and higher risk of hospitalization at 1-, 3-, and 5-years post LRTI, but differences diminished with time. Secondly, older adults experiencing LRTIs had higher total number of hospital days post-LRTI episode, even if the associations gradually weakened. Thirdly, LRTIs increased the rate of visits to secondary and tertiary healthcare services. Lastly, the stratified analyses revealed differences in the studied associations by sex, age and obesity.

### Comparison with other studies

In accordance with previous investigations [3, 14], males exhibited higher rates of extended hospitalization, as well as 1-, 3-, and 5-year hospitalization and mortality compared to females. Notably, several studies consistently found that males have longer hospital stays than females, regardless of the reason for admission, and this observation may shed light on the sex disparities identified in our study [29, 30]. A study conducted in Sweden in 2016 confirmed the trend of men predominantly referred to high-intensity specialized care [28]. Furthermore, prior research has indicated elevated immediate mortality rates among males following LRTIs [14]. These disparities in healthcare utilization and mortality between sexes may arise from multifaceted factors, including pre-infection socioeconomic status, sex-specific healthcare needs, and lifestyle behaviors [4, 11, 28, 29]. Although males are often predominant in resource-demanding specialist care, there is a growing acknowledgment of the importance of sex- and gender-sensitive healthcare policies and interventions [30].

Our results show that post-LRTI, older adults between the ages of 60 and 75 had a higher rate of days spent in the hospital compared to older adults aged 75 and above. One possible explanation for the shorter hospital stays among adults aged 75 and above is the higher observed mortality in this group (including potential in-hospital deaths). Additionally, clinical recommendations and concerns about the potential risks associated with hospital admissions for the oldest old adults may also play a role. These concerns are related to the higher vulnerability of the oldest old adults to complications that can arise during hospitalization, such as hospital-acquired infections, deep vein thrombosis, increased disability, and worsening of existing medical conditions [31–33]. These complications are known to be associated with longer hospital stays and higher mortality rates [34]. Consequently, healthcare providers may exercise caution when admitting the oldest old adults to hospitals for prolonged periods.

Regarding the moderating effects of obesity, we found a higher risk of hospitalization at 1-, 3-, and 5-years following LRTI in obese vs. non-obese participants. Biological reasons that could explain the observed increased risk among obese participants include obesity-induced chronic low-grade inflammation and decreased immune system response [35, 36]. This is coupled by altered respiratory mechanics such as excess fat tissue in the chest and abdomen affecting breathing, reduced lung volume, reduce muscle strength, and mechanical compressions on the diaphragm and chest cavity [37–39]. Moreover, obesity is associated with metabolic dysfunction [35] and different co-morbidities that increase the risk of hospitalization, mortality, and medications utilization [40–42]. Obesity has also been linked to poorer response to different vaccines, such as for H1N1 [43] and COVID-19 [44], warranting life style modifications to potentially reduce these risks.

### Public health implications

The COVID-19 pandemic has highlighted the significant burden that respiratory infections, including LRTIs, can impose on healthcare systems and vulnerable populations [45]. The global response to COVID-19 offers valuable lessons applicable to addressing LRTIs, given the similarities in outcomes. The increased risk of mortality following LRTIs, the higher risks of hospitalization, and the impact on length of stay and healthcare visits substantiate the burden on healthcare resources and the need for effective interventions to reduce the incidence and severity of LRTIs in older adults. Public health policies should emphasize the importance of comprehensive care for older adults, including preventive measures (e.g., promoting vaccination against respiratory infections), early diagnosis, appropriate treatment, sex-sensitive healthcare policies and post-hospitalization care to improve overall health outcomes [46–48].

### Strengths and limitations

The study boasts several notable strengths. Firstly, it draws upon data from the Swedish National Study of Aging and Care in Kungsholmen (SNAC-K), a prospective cohort study, with a big sample of older (including oldest old) adults. Additionally, the extended follow-up duration provides valuable insights into the long-term consequences of LRTIs in older adults. Moreover, the comprehensive data collection within the SNAC-K study enables accounting for several relevant cofounders. Information from the Swedish National Patient Register and Cause of Death Register bolsters the study's internal validity by relying on robust outcome data.

However, several limitations warrant acknowledgment. The SNAC-K cohort is located in a wealthy part of Stockholm, with a higher number of participants with university-level education compared to similar age Swedish older adults. Despite efforts to address confounders through propensity score-based matching with a comprehensive set of covariates followed by inverse probably weighting, statistical methods employed may not be able to account for residual and unmeasured confounding, which still remain possible. The study's focus on specific healthcare usage outcomes may have overlooked other important aspects of LRTIs, such as functional outcomes. Additionally, the lack of data on hospitalizations previous to the index date, and data on pneumonia etiology, severity, and treatment limit us from a comprehensive understanding of contributing factors. Furthermore, the study's exclusion of arguably milder LRTI cases managed in primary care may limit generalizability, and the results may not fully reflect the experiences of individuals with less severe cases of LRTIs.

## Conclusions

In conclusion, our study provides insights into the significant impact of LRTIs on older adults' health outcomes and healthcare utilization. Stratified analyses also indicated differences based on sex, age, and obesity status, with males exhibiting higher risks overall. The findings highlight the need for preventive strategies and targeted interventions to reduce the burden of LRTIs in this vulnerable population. Moreover, the findings underline the need for healthcare systems to anticipate and plan for the increased demand of healthcare resources among older adults exposed to LRTIs. Further research is warranted to explore additional factors and potential interventions that could mitigate the impact of LRTI on health outcomes and healthcare utilization.

### Supplementary Information

Below is the link to the electronic supplementary material.

Supplementary file 1 (DOCX 57 KB)

Supplementary file 2 (DOCX 35 KB)

## Data Availability

The datasets generated and/or analyzed during the current study are not publicly available due to ethical and data sharing restrictions/laws, including but not limited to GDPR. The data, however, can be requested formally from the SNAC-K principal investigator.
