# Supplementary material for: Impact of lower-respiratory tract infections on healthcare utilization and mortality in older adults: a Swedish population-based cohort study
Source: Aging Clin Exp Res. 2024 Jul 17;36(1):146. doi: 10.1007/s40520-024-02808-5 (PMC11254993; doi:10.1007/s40520-024-02808-5)
Supplement: Supplementary file 1 — (DOCX 57 KB) [file 40520_2024_2808_MOESM1_ESM.docx]

**Impact of lower-respiratory tract infections on healthcare utilization and mortality in older adults: a Swedish population-based cohort study**

**Supplementary File 1**

**Journal: Aging Clinical and Experimental Research**

**Authors:**

Ahmad Abbadi^1,2^, Susanna Gentili^1^, Eleana Tsoumani^3^, Agnes Brandtmüller^4^, Merle K Hendel^1^, Stina Salomonsson^5^, Amaia Calderón-Larrañaga^1,6^, Davide L. Vetrano^1,6^

**Affiliations:**

1. Aging Research Center, Department of Neurobiology, Care Sciences and Society, Karolinska Institutet and Stockholm University, Stockholm, Sweden

2. Department of Medical Epidemiology and Biostatistics, Karolinska Institutet, Stockholm,

Sweden

3. Center for Observational and Real-World Evidence, MSD, Athens, Greece

4. Center for Observational and Real-World Evidence, MSD, Budapest, Hungary

5. Center for Observational and Real-World Evidence, MSD, Stockholm, Sweden

6. Stockholm Gerontology Research Center, Stockholm, Sweden

**Corresponding author:**

Ahmad Abbadi, MD MMedSc

Department of Medical Epidemiology and Biostatistics, Karolinska Institutet

Nobels väg 12A, 171 65 Solna, Sweden

[ahmad.abbadi@ki.se](mailto:ahmad.abbadi@ki.se)

**Supplementary Material:**

Table S1. Identification of exposure and outcomes.

| Exposure Identification | |
| --- | --- |
| ICD-10 Code | Description |
| J09 | Influenza due to certain identified influenza viruses |
| J10 | Influenza due to other identified influenza virus |
| J11 | Influenza due to unidentified influenza virus |
| J12 | Viral pneumonia, not elsewhere classified |
| J13 | Pneumonia due to Streptococcus pneumoniae |
| J14 | Pneumonia due to Hemophilus influenzae |
| J15 | Bacterial pneumonia, not elsewhere classified |
| J16 | Pneumonia due to other infectious organisms, not elsewhere classified |
| J17 | Pneumonia in diseases classified elsewhere |
| J18 | Pneumonia, unspecified organism |
| J20 | Acute bronchitis |
| J21 | Acute bronchiolitis |
| J22 | Unspecified acute lower respiratory infection |
| Outcome Identification | Description and source |
| All-cause mortality | Death documented in the Swedish Cause of Death register (up to 19-years follow-up) |
| Hospitalization | Any admission to the hospital (yes/no), excluding hospitalization during the LRTI episode, documented in the Swedish Inpatient Register. Presented in 1-, 3-, and 5-years hospitalization. |
| Days stayed in inpatient hospital admissions | length of stay in days in hospitals during an admission. Documented in the Swedish Inpatient Register, and excluded the hospitalization during the LRTI episode. |
| Number of hospital admissions | Count of hospital admissions (summation of total number), excluding the hospitalization during LRTI episode. Documented in the Swedish Inpatient Register. |
| Number of specialized outpatient care visits | Count of specialized outpatient clinic visits (summation of total number), excluding the diagnosis/treatment visit during LRTI episode. Documented in the Swedish Outpatient Register. |
| Total healthcare visits | Count of both hospital admissions and specialized outpatient clinic visits (summation of total number). Excludes the hospitalization/outpatient visit during the LRTI episode. |

Table S2. Supplement to the hazard ratios of mortality following LRTIs.

|  | **IPW- weighted HR** | **95% CI** |
| --- | --- | --- |
| ***Overall*** |  |  |
| *RCS1* | 1.58** | (1.31, 1.90) |
| *RCS2* | 0.91** | (0.89, 0.92) |
| *RCS3* | 0.99 | (0.97, 1.01) |
| *RCS4* | 1.00 | (0.99, 1.01) |
| *RCS5* | 1.00 | (0.99, 1.01) |
| *constant* | 1.62* | (1.06, 2.49) |
| ***Males*** |  |  |
| *RCS1* | 1.01** | (1.01, 1.02) |
| *RCS2* | 0.99** | (0.99, 1.00) |
| *RCS3* | 1.00** | (1.00, 1.00) |
| *RCS4* | 1.00 | (1.00, 1.00) |
| *RCS5* | 1.00 | (1.00, 1.00) |
| *constant* | 58.16** | (58.05, 58.26) |
| ***Females*** |  |  |
| *RCS1* | 1.53 | (0.30, 7.79) |
| *RCS2* | 0.90* | (0.82, 0.99) |
| *RCS3* | 0.99 | (0.85, 1.14) |
| *RCS4* | 1.00 | (0.97, 1.04) |
| *RCS5* | 1.00 | (0.98, 1.01) |
| *constant* | 1.78 | (0.03, 106.47) |
| ***Age <75*** |  |  |
| *RCS1* | 1.38* | (1.08, 1.77) |
| *RCS2* | 1.00 | (0.92, 1.08) |
| *RCS3* | 0.97 | (0.94, 1.01) |
| *RCS4* | 0.99 | (0.97, 1.01) |
| *RCS5* | 0.98 | (0.96, 1.00) |
| *constant* | 0.63 | (0.32, 1.24) |
| ***Age ≥75*** |  |  |
| *RCS1* | 1.04 | (0.79, 1.38) |
| *RCS2* | 0.99 | (0.92, 1.06) |
| *RCS3* | 1.00 | (0.98, 1.02) |
| *RCS4* | 1.00 | (0.99, 1.00) |
| *RCS5* | 1.00 | (0.99, 1.00) |
| *constant* | 19.66 | (0.03, 12042.33) |
| ***No Obesity*** |  |  |
| *RCS1* | 1.15 | (0.48, 2.75) |
| *RCS2* | 0.95 | (0.73, 1.24) |
| *RCS3* | 0.98 | (0.93, 1.04) |
| *RCS4* | 1.00 | (0.99, 1.00) |
| *RCS5* | 1.00 | (0.99, 1.00) |
| *constant* | 6.04 | (0.01, 4848.71) |
| ***Obesity*** |  |  |
| *RCS1* | 1.00** | (1.00, 1.00) |
| *RCS2* | 1.00** | (1.00, 1.00) |
| *RCS3* | 1.00** | (1.00, 1.00) |
| *RCS4* | 1.00* | (1.00, 1.00) |
| *RCS5* | 1.00 | (1.00, 1.00) |
| *constant* | 310.02** | (309.91, 310.14) |

Table S3. Supplement to the hazard ratios of hospitalization following LRTIs.

|  | **IPW- weighted HR** | **95% CI** |  |
| --- | --- | --- | --- |
| **1-year hospitalization** | | |  |
| ***Overall*** |  |  |  |
| *RCS1* | 2.33** | (2.15, 2.53) |  |
| *RCS2* | 1.04 | (0.99, 1.09) |  |
| *RCS3* | 1.00 | (0.97, 1.03) |  |
| *RCS4* | 0.99 | (0.98, 1.01) |  |
| *RCS5* | 1.00 | (0.99, 1.01) |  |
| *constant* | 0.17** | (0.15, 0.19) |  |
| ***Males*** |  |  |  |
| *RCS1* | 2.75** | (2.44, 3.10) |  |
| *RCS2* | 1.08 | (1.00, 1.17) |  |
| *RCS3* | 1.02 | (0.97, 1.07) |  |
| *RCS4* | 0.99 | (0.96, 1.03) |  |
| *RCS5* | 1.00 | (0.99, 1.02) |  |
| *constant* | 0.17** | (0.14, 0.21) |  |
| ***Females*** |  |  |  |
| *RCS1* | 2.12** | (1.92, 2.23) |  |
| *RCS2* | 1.02 | (0.97, 1.08) |  |
| *RCS3* | 0.98 | (0.95, 1.01) |  |
| *RCS4* | 0.99 | (0.98, 1.01) |  |
| *RCS5* | 1.00 | (0.99, 1.01) |  |
| *constant* | 0.16** | (0.14, 0.19) |  |
| ***Age <75*** |  |  |  |
| *RCS1* | 1.77** | (1.60, 1.95) |  |
| *RCS2* | 1.08** | (1.03, 1.13) |  |
| *RCS3* | 1.00 | (0.97, 1.03) |  |
| *RCS4* | 0.99 | (0.97, 1.01) |  |
| *RCS5* | 1.00 | (0.99, 1.02) |  |
| *constant* | 0.11** | (0.09, 0.13) |  |
| ***Age ≥75*** |  |  |  |
| *RCS1* | 2.62** | (2.36, 3.04) |  |
| *RCS2* | 1.01 | (0.95, 1.07) |  |
| *RCS3* | 0.99 | (0.96, 1.03) |  |
| *RCS4* | 0.99 | (0.97, 1.02) |  |
| *RCS5* | 1.00 | (0.99, 1.01) |  |
| *constant* | 0.19** | (0.16, 0.22) |  |
| ***No Obesity*** |  |  |  |
| *RCS1* | 2.34** | (2.14, 2.55) |  |
| *RCS2* | 1.02 | (0.98, 1.07) |  |
| *RCS3* | 1.00 | (0.97, 1.04) |  |
| *RCS4* | 0.99 | (0.98, 1.01) |  |
| *RCS5* | 1.00 | (0.99, 1.01) |  |
| *constant* | 0.18** | (0.15, 0.20) |  |
| ***Obesity*** |  |  |  |
| *RCS1* | 2.37** | (1.80, 3.12) |  |
| *RCS2* | 1.17 | (0.85, 1.60) |  |
| *RCS3* | 1.00 | (0.89, 1.12) |  |
| *RCS4* | 0.98 | (0.94, 1.01) |  |
| *RCS5* | 1.00 | (0.98, 1.02) |  |
| *constant* | 0.12** | (0.09, 0.17) |  |
| **3-years hospitalization** | | | |
| ***Overall*** | |  |  |
| *RCS1* | | 3.04** | (2.81, 2.07) |
| *RCS2* | | 1.04 | (0.98, 1.10) |
| *RCS3* | | 0.98 | (0.95, 1.01) |
| *RCS4* | | 1.00 | (0.99, 1.02) |
| *RCS5* | | 1.00 | (0.99, 1.01) |
| *constant* | | 0.30** | (0.27, 0.32) |
| ***Males*** | |  |  |
| *RCS1* | | 3.44** | (3.06, 3.88) |
| *RCS2* | | 1.16** | (1.04, 1.28) |
| *RCS3* | | 1.00 | (0.94, 1.07) |
| *RCS4* | | 1.00 | (0.97, 1.03) |
| *RCS5* | | 1.00 | (0.98, 1.01) |
| *constant* | | 0.28** | (0.25, 0.32) |
| ***Females*** | |  |  |
| *RCS1* | | 2.86** | (2.59, 3.16) |
| *RCS2* | | 0.98 | (0.92, 1.04) |
| *RCS3* | | 0.96* | (0.93, 0.99) |
| *RCS4* | | 1.01 | (0.99, 1.03) |
| *RCS5* | | 1.00 | (0.99, 1.01) |
| *constant* | | 0.30** | (0.27, 0.34) |
| ***Age <75*** | |  |  |
| *RCS1* | | 2.26** | (2.04, 2.51) |
| *RCS2* | | 1.06 | (0.99, 1.13) |
| *RCS3* | | 0.96* | (0.92, 0.99) |
| *RCS4* | | 0.99 | (0.97, 1.01) |
| *RCS5* | | 1.00 | (0.99, 1.01) |
| *constant* | | 0.22** | (0.19, 0.26) |
| ***Age ≥75*** | |  |  |
| *RCS1* | | 3.45** | (3.12, 3.82) |
| *RCS2* | | 1.00 | (0.93, 1.08) |
| *RCS3* | | 0.98 | (0.94, 1.02) |
| *RCS4* | | 1.01 | (0.99, 1.03) |
| *RCS5* | | 1.00 | (0.99, 1.01) |
| *constant* | | 0.31** | (0.28, 0.35) |
| ***No Obesity*** | |  |  |
| *RCS1* | | 3.05** | (2.81, 3.32) |
| *RCS2* | | 1.03 | (0.97, 1.09) |
| *RCS3* | | 0.99 | (0.95, 1.02) |
| *RCS4* | | 1.00 | (0.98, 1.02) |
| *RCS5* | | 1.00 | (0.99, 1.01) |
| *constant* | | 0.30** | (0.28, 0.33) |
| ***Obesity*** | |  |  |
| *RCS1* | | 3.07** | (2.39, 3.93) |
| *RCS2* | | 1.21 | (0.93, 1.58) |
| *RCS3* | | 0.91 | (0.82, 1.02) |
| *RCS4* | | 1.03 | (0.98, 1.08) |
| *RCS5* | | 1.02 | (0.99, 1.04) |
| *constant* | | 0.26** | (0.21, 0.32) |
| **5-years hospitalization** | | | |
| ***Overall*** | |  |  |
| *RCS1* | | 3.35** | (3.11, 3.62) |
| *RCS2* | | 1.04 | (0.98, 1.10) |
| *RCS3* | | 0.98 | (0.95, 1.01) |
| *RCS4* | | 1.01 | (0.99, 1.03) |
| *RCS5* | | 1.00 | (0.99, 1.01) |
| *constant* | | 0.35** | (0.33, 0.38) |
| ***Males*** | |  |  |
| *RCS1* | | 3.78** | (3.37, 4.24) |
| *RCS2* | | 1.15** | (1.05, 1.27) |
| *RCS3* | | 0.98 | (0.92, 1.05) |
| *RCS4* | | 0.99 | (0.96, 1.02) |
| *RCS5* | | 1.00 | (0.98, 1.01) |
| *constant* | | 0.33** | (0.30, 0.38) |
| ***Females*** | |  |  |
| *RCS1* | | 3.17** | (2.88, 3.49) |
| *RCS2* | | 0.98 | (0.92, 1.03) |
| *RCS3* | | 0.98 | (0.95, 1.01) |
| *RCS4* | | 1.02 | (1.00, 1.05) |
| *RCS5* | | 1.00 | (0.99, 1.02) |
| *constant* | | 0.36** | (0.33, 0.39) |
| ***Age <75*** | |  |  |
| *RCS1* | | 2.55** | (2.30, 2.84) |
| *RCS2* | | 1.04 | (0.97, 1.11) |
| *RCS3* | | 0.95** | (0.91, 0.98) |
| *RCS4* | | 1.01 | (0.99, 1.04) |
| *RCS5* | | 1.01 | (0.99, 1.02) |
| *constant* | | 0.30** | (0.27, 0.34) |
| ***Age ≥75*** | |  |  |
| *RCS1* | | 3.77** | (3.42, 4.17) |
| *RCS2* | | 1.00 | (0.93, 1.07) |
| *RCS3* | | 0.98 | (0.94, 1.03) |
| *RCS4* | | 1.01 | (0.98, 1.03) |
| *RCS5* | | 1.00 | (0.98, 1.01) |
| *constant* | | 0.35** | (0.32, 0.39) |
| ***No Obesity*** | |  |  |
| *RCS1* | | 3.37** | (3.10, 3.66) |
| *RCS2* | | 1.03 | (0.97, 1.09) |
| *RCS3* | | 0.99 | (0.96, 1.02) |
| *RCS4* | | 1.00 | (0.98, 1.02) |
| *RCS5* | | 1.01 | (1.00, 1.02) |
| *constant* | | 0.36** | (0.33, 0.38) |
| ***Obesity*** | |  |  |
| *RCS1* | | 3.33** | (2.71, 4.10) |
| *RCS2* | | 1.14 | (0.94, 1.37) |
| *RCS3* | | 0.91 | (0.83, 1.00) |
| *RCS4* | | 1.04 | (0.99, 1.09) |
| *RCS5* | | 0.98 | (0.95, 1.01) |
| *constant* | | 0.32** | (0.26, 0.39) |

Table S4. Overall rate of days stayed in inpatient hospital admissions among those exposed and unexposed to LRTIs*.*

|  | **IRR** | **95% CI** |
| --- | --- | --- |
| ***Males*** |  |  |
| No LRTI | Ref | Ref |
| LRTI | **1.79** | (1.41; 2.27) |
| ***Females*** |  |  |
| No LRTI | Ref | Ref |
| LRTI | 1.20 | (0.94; 1.53) |
| ***Age <75*** |  |  |
| No LRTI | Ref | Ref |
| LRTI | **1.80** | (1.27; 2.55) |
| ***Age ≥75*** |  |  |
| No LRTI | Ref | Ref |
| LRTI | **1.27** | (1.10; 1.48) |
| ***No Obesity*** |  |  |
| No LRTI | Ref | Ref |
| LRTI | **1.40** | (1.16; 1.69) |
| ***Obesity*** |  |  |
| No LRTI | Ref | Ref |
| LRTI | 1.36 | (0.94; 1.98) |

Table S5. One-year rate of days stayed in inpatient hospital admissions among those exposed and unexposed to LRTIs*.*

|  | **IRR** | **95% CI** |
| --- | --- | --- |
| ***Males*** |  |  |
| No LRTI | Ref | Ref |
| LRTI | **3.58** | (2.37; 5.40) |
| ***Females*** |  |  |
| No LRTI | Ref | Ref |
| LRTI | **1.39** | (1.00; 1.94) |
| ***Age <75*** |  |  |
| No LRTI | Ref | Ref |
| LRTI | **4.25** | (2.42; 7.45) |
| ***Age ≥75*** |  |  |
| No LRTI | Ref | Ref |
| LRTI | **1.90** | (1.45; 2.48) |
| ***No Obesity*** |  |  |
| No LRTI | Ref | Ref |
| LRTI | **2.00** | (1.50; 2.65) |
| ***Obesity*** |  |  |
| No LRTI | Ref | Ref |
| LRTI | **3.35** | (1.50; 7.47) |

Table S6. Three-year rate of days stayed in inpatient hospital admissions among those exposed and unexposed to LRTIs*.*

|  | **IRR** | **95% CI** |
| --- | --- | --- |
| ***Males*** |  |  |
| No LRTI | Ref | Ref |
| LRTI | **2.23** | (1.61; 3.10) |
| ***Females*** |  |  |
| No LRTI | Ref | Ref |
| LRTI | **1.31** | (1.01; 1.69) |
| ***Age <75*** |  |  |
| No LRTI | Ref | Ref |
| LRTI | **2.94** | (1.97; 4.40) |
| ***Age ≥75*** |  |  |
| No LRTI | Ref | Ref |
| LRTI | **1.54** | (1.26; 1.88) |
| ***No Obesity*** |  |  |
| No LRTI | Ref | Ref |
| LRTI | **1.58** | (1.27; 1.97) |
| ***Obesity*** |  |  |
| No LRTI | Ref | Ref |
| LRTI | **1.98** | (1.14; 3.43) |

*Table S7. Five-year* rate of days stayed in inpatient hospital admissions among those exposed and unexposed to LRTIs*.*

|  | **IRR** | **95% CI** |
| --- | --- | --- |
| ***Males*** |  |  |
| No LRTI | Ref | Ref |
| LRTI | **2.08** | (1.56; 2.77) |
| ***Females*** |  |  |
| No LRTI | Ref | Ref |
| LRTI | 1.16 | (0.91; 1.47) |
| ***Age <75*** |  |  |
| No LRTI | Ref | Ref |
| LRTI | **2.12** | (1.48; 3.03) |
| ***Age ≥75*** |  |  |
| No LRTI | Ref | Ref |
| LRTI | **1.52** | (1.26; 1.83) |
| ***No Obesity*** |  |  |
| No LRTI | Ref | Ref |
| LRTI | **1.42** | (1.17; 1.73) |
| ***Obesity*** |  |  |
| No LRTI | Ref | Ref |
| LRTI | **1.87** | (1.13; 3.11) |

*Table S8. Total healthcare visits* among those exposed and unexposed to LRTIs*.*

|  | **IRR** | **95% CI** |
| --- | --- | --- |
| ***Males*** |  |  |
| No LRTI | Ref | Ref |
| LRTI | **1.62** | (1.29; 2.04) |
| ***Females*** |  |  |
| No LRTI | Ref | Ref |
| LRTI | **1.38** | (1.13; 1.69) |
| ***Age <75*** |  |  |
| No LRTI | Ref | Ref |
| LRTI | **1.56** | (1.29; 1.88) |
| ***Age ≥75*** |  |  |
| No LRTI | Ref | Ref |
| LRTI | 1.14 | (0.97; 1.36) |
| ***No Obesity*** |  |  |
| No LRTI | Ref | Ref |
| LRTI | **1.43** | (1.22; 1.66) |
| ***Obesity*** |  |  |
| No LRTI | Ref | Ref |
| LRTI | **1.76** | (1.08; 2.85) |

*Table S9. Total specialized outpatient care visits* among those exposed and unexposed to LRTIs*.*

|  | **IRR** | **95% CI** |
| --- | --- | --- |
| ***Males*** |  |  |
| No LRTI | Ref | Ref |
| LRTI | **1.59** | (1.23; 2.06) |
| ***Females*** |  |  |
| No LRTI | Ref | Ref |
| LRTI | **1.39** | (1.12; 1.73) |
| ***Age <75*** |  |  |
| No LRTI | Ref | Ref |
| LRTI | **1.54** | (1.24; 1.92) |
| ***Age ≥75*** |  |  |
| No LRTI | Ref | Ref |
| LRTI | 1.07 | (0.89; 1.29) |
| ***No Obesity*** |  |  |
| No LRTI | Ref | Ref |
| LRTI | **1.41** | (1.33; 1.68) |
| ***Obesity*** |  |  |
| No LRTI | Ref | Ref |
| LRTI | **1.78** | (1.05; 3.02) |

*Table S10. Total hospital admissions* among those exposed and unexposed to LRTIs*.*

|  | **IRR** | **95% CI** |
| --- | --- | --- |
| ***Males*** |  |  |
| No LRTI | Ref | Ref |
| LRTI | **1.85** | (1.50; 2.29) |
| ***Females*** |  |  |
| No LRTI | Ref | Ref |
| LRTI | **1.41** | (1.13; 1.76) |
| ***Age <75*** |  |  |
| No LRTI | Ref | Ref |
| LRTI | **1.69** | (1.27; 2.24) |
| ***Age ≥75*** |  |  |
| No LRTI | Ref | Ref |
| LRTI | **1.56** | (1.30; 1.86) |
| ***No Obesity*** |  |  |
| No LRTI | Ref | Ref |
| LRTI | **1.57** | (1.32; 1.86) |
| ***Obesity*** |  |  |
| No LRTI | Ref | Ref |
| LRTI | **1.54** | (1.05; 2.26) |
